# Prediction of the Size of the Fragment in Comminuted Coronoid Fracture Using the Contralateral Side: An Analysis of Similarity of Bilateral Ulnar Coronoid Morphology

**DOI:** 10.1111/os.12780

**Published:** 2020-10-05

**Authors:** Hai‐long Zhang, Kun‐Jhih Lin, Yi Lu

**Affiliations:** ^1^ Department of Sports Medicine Beijing Jishuitan Hospital Beijing China; ^2^ Department of Electrical Engineering & Translation Technology Center for Medical Device Chung Yuan Christian University Taoyuan China

**Keywords:** Bilateral, Comparison, Coronoid process, Morphology

## Abstract

**Objective:**

To evaluate the morphological similarity of bilateral coronoid process.

**Methods:**

A total of 128 sets of computed tomography images of bilateral coronoid process from patients between January 2015 and December 2016 were acquired for three‐dimensional reconstruction to generate a coronoid process model. The patients were aged between 31.4 ± 9.3 years. The upper 40% of the coronoid process was trimmed as targeted fragment for morphological analysis. The height, length, width as well as the radius of the medial and lateral facet of the targeted fragment were compared in terms of laterality, age, and gender. To evaluate the similarity of the articular surface of the coronoid process, a local coordinate was created and coordinate transformation algorithm was developed to realign the bilateral coronoid process for the following matching. Then Delaunay triangulation was introduced for calculation of the area of the articular surface. After matching of articular surface of the upper 40% of bilateral coronoid process, the overlapping area of the articular surface was quantified to assess the similarity in morphology and compared in regard to age and gender.

**Results:**

In this study, the height of the target fragment was 12.40 ± 2.74 mm, which was 12.62 ± 2.06 mm for male patients and 12.13 ± 3.76 mm for female patients (*t* = 0.94, *P* = 0.35). The height of the target fragment was 12.79 ± 1.76 mm for patients >40 years and 13.23 ± 3.16 mm for patients <40 years (*t* = 1.11, *P* = 0.27). The height of the target fragment of left and right coronoid process was 12.26 ± 3.40 mm and 12.74 ± 2.79 mm (*t* = 1.15, *P* = 0.25). The length of the target fragment was 23.81 ± 2.67 mm, which was 23.86 ± 2.11 mm for male patients and 23.76 ± 2.85 mm for female patients (*t* = 0.23, *P* = 0.82). The length of the target fragment was 22.92 ± 1.96 mm for patients >40 years and 23.23 ± 2.14 mm for patients <40 years (*t* = 0.76, *P* = 0.45). The length of the target fragment of left and right coronoid process was 22.52 ± 2.89 mm and 21.66 ± 3.01 mm, respectively (*t* = 1.00, *P* = 0.32). The width of the target fragment was 23.12 ± 1.92 mm on average, which was 23.06 ± 1.54 mm for male patients and 23.19 ± 2.82 mm for female patients (*t* = 0.33, *P* = 0.74). The width of the target fragment was 24.82 ± 2.23 mm for patients >40 years and 23.46 ± 3.38 mm for patients <40 years (*t* = 1.56, *P* = 0.12). The width of target fragment of left and right coronoid process was 24.42 ± 2.22 mm and 24.47 ± 2.69 mm, respectively (*t* = 1.31, *P* = 0.19). The radius of medial facet was 6.44 ± 1.01 mm, which was 6.41 ± 1.39 mm for male patients and 6.47 ± 0.95 mm for female patients (*t* = 0.28, *P* = 0.78). The radius of medial facet was 6.82 ± 1.28 mm for patients >40 years and 6.46 ± 0.94 mm for patients <40 years (*t* = 1.31, *P* = 0.19). The radius of medial facet of left and right coronoid process was 6.43 ± 1.24 mm and 6.64 ± 1.34 mm (*t* = 1.60, *P* = 0.11). The radius of lateral facet was 11.84 ± 3.71 mm, which was 11.61 ± 4.24 mm for male patients and 12.11 ± 3.09 mm for female patients (*t* = 0.74, *P* = 0.46). The radius of medial facet was 11.82 ± 3.28 mm for patients >40 years and 12.46 ± 3.94 mm for patients <40 years (*t* = 1.02, *P* = 0.31). The radius of lateral facet of left and right coronoid process was 11.97 ± 5.31 mm and 10.29 ± 3.29 mm, respectively (*t* = 1.70, *P* = 0.09). The covering percentage of the articular surface of the upper 40% of bilateral coronoid process was 87% ± 12% with the covering percentage as 85.3% ± 14.2% for male patients and 90.0% ± 11.2% for female patients (*t* = 0.75, *P* = 0.41). The covering percentage was 88.2% ± 11.7% for patients >40 years and it was 87.4% ± 13.2% for patients <40 years (*t* = 0.98, *P* = 0.33).

**Conclusions:**

The present study suggested that bilateral coronoid process shares high similarity in terms of 3D structure and articular surface morphology, which suggested that the osseous architecture of the coronoid process with comminuted fracture could be predicted by the morphological information of the contralateral side.

## Graphical abstract

Coronoid is a crucial anatomical structure in maintaining elbow function and reconstruction of coronoid is of great importance in restoration of elbow stability. Zhang, et al. revealed the similarity of bilateral coronoid in morphology, which suggested an option of restoration of coronoid defect with three‐dimensional printed prosthesis based on the spatial information of the contralateral coronoid

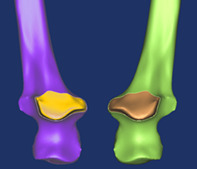

## Introduction

The coronoid process of the ulna plays an important role in the stability of the elbow joint, and biomechanical evidence suggests it functions as a stabilizer in preventing posterior dislocation and resisting rotational instability^1^, ^2^, ^3^. Fracture of the coronoid is commonly related to terrible triad and other complex injuries. Traditionally, coronoid fractures can be classified into three types based on fragment height relative to the total height of the coronoid^2^. Type I fractures involve the tip of the coronoid, type II fractures involve 40% or less of coronoid height, and fracture comprising greater than 40% of the coronoid process is classified as type III. Both clinical and biomechanical studies have suggested that repair of larger coronoid fractures is crucial to restore elbow function, avoid posterior elbow dislocation, and prevent subsequent post‐traumatic arthritis. If left untreated, it may result in persistent instability of elbow and lost function in daily life activity^4^.

Currently, open reduction and internal fixation have been suggested as the primary treatment choice for restoration of the elbow joint stability, but if the fracture is not amenable to fixation due to severity of fracture pattern, biological reconstruction or prosthetic replacement is required^5^, ^6^, ^7^. To date, numerous methods have been developed for biological reconstruction of coronoid process, including olecranon tip, iliac crest, radial head, rib or navicular^8^, ^9^, ^10^, ^11^, ^12^ autograft, or allograft. Although these bone graft techniques achieved good or fair results in some patients in the early term follow‐up, this concept has not gained popularity due to a lack of long term follow‐up result and the donor site comorbidity^13^, ^14^, ^15^. A possible explanation is that the coronoid articular surface articulates with the trochlea of the humerus with a convex lateral surface and concave medial surface. The convex surface appears to match the articular facet of the olecranon, whereas the concave surface matches the radial head. Because of complex geometry of the articular surface of the coronoid process, none of the above‐mentioned grafts can exactly reconstruct the surface contour. Replacement of the coronoid process with prosthesis is another option for restoration of the elbow joint stability, but it is only performed in very limited cases and outcome is not as expected^7^. O'Driscoll *et al*. developed a coronoid prosthesis and reported its primary clinical outcome^7^. The prosthesis was designed to replace 40% of the height of the coronoid because that height closely approximates the mean height of the terrible‐triad coronoid fracture, and biomechanical testing has indicated the prosthesis could function in resisting dislocation and rotational instability^3^. Their report suggested its application could reduce pain and restore stability, but not restore the anatomy and full function of the elbow^7^.

From a practical perspective, a clinically successful coronoid prosthesis will need to meet the requirements from an excellent anatomic conformity with the trochlea and simplicity of design, which balance the clinical outcome and the economic cost. The disadvantages are that the anatomic conformity will require an exact replication of the anatomy and potentially greater range of size and shape choices. A simpler design will compromise on conformity and result in poor pressure distribution on the trochlea, which would potentially accelerate cartilage degeneration on the trochlea. Also, as the stability was maintained partially by the conformity, poor conformity would result in minor instability which would lead to loosening of the prosthesis and affect the longevity of the prosthesis. Thus, it will be important to precisely determine the original coronoid shape to rebuild.

Accurate assessment of the size and geometric characteristics of the coronoid is paramount as it plays the main role in the longevity of the prothesis^6^, ^7^. Currently, this question has still not been fully addressed, especially the joint conformity, which needs to be restored in a patient‐specific manner; this means one possible solution to achieve a perfect balance between conformity and simplicity would be to have each coronoid prosthesis custom made by three‐dimensional (3D) printing. As the bony structure of the coronoid process has been significantly compromised as a result of the comminuted fracture, difficulty would be encountered when the spatial information was subtracted for 3D printing. Previous studies have suggested a high symmetry of osseous architecture of bilateral limbs, and evaluation of the pathology in one limb using the contralateral structure as a mirror‐image template has resulted in satisfactory outcome^16^, ^17^. Thus, it offers a potential option to reconstruct the 3D model of the comminuted coronoid process using the structural information from the contralateral one. Unfortunately no studies have been carried out to assess the morphological similarity of the coronoid process by comparison with the contralateral side.

The purpose of the present study was to evaluate the morphological similarity of the coronoid process compared to the contralateral side using computed tomography (CT) scan, which included: (i) comparison of the parameters bilaterally; (ii) comparison of the parameters between different age group; and (iii) comparison of the parameters between different gender groups. To our knowledge, this is the first study to quantitatively compare the morphological similarity of bilateral coronoid process.

## Patients and Methods

### *Inclusion and Exclusion Criteria*

Patients were selected using the following inclusion criteria: (i) patient diagnosed as soft tissue injury around elbow; (ii) underwent CT scan of elbow; (iii) CT scan of the contralateral normal side elbow was also acquired; (iv) 3D model of coronoid process were reconstructed; (v) an experimental study. Patients were excluded if they were associated with pathology of the proximal ulna, such as: (i) fracture; (ii) deformity; (iii) osteoarthritis; (iv) bone degeneration. Body mass index were not taken into consideration in this study.

### *Study Design*

The study was ethically approved by the institutional review board, and informed consent was obtained from all the patients. A total of 128 patients (70 male and 58 female patients) from January 2015 to December 2016 were enrolled and the age was 31.4 ± 9.3 years.

### *Image Acquisition and* *3D CT* *Model*

CT (Aquillion 64, Toshiba America Medical Systems, Tustin, CA) scans of bilateral elbows of all the patients were acquired. The CT scans were performed with a resolution of 512 × 512 pixels and a slice thickness of 0.8 mm using the standard 120 kVp and 200 mA bone reconstruction sequence. The 3D geometric model of the proximal ulna was reconstructed from the CT scan images and the surface of the model was exported in point cloud format using commercial software (Mimics, Materialize Inc., Leuven, Belgium).

### *Measurement of Anatomical Parameters of Coronoid Process*

**Fig. 1:**
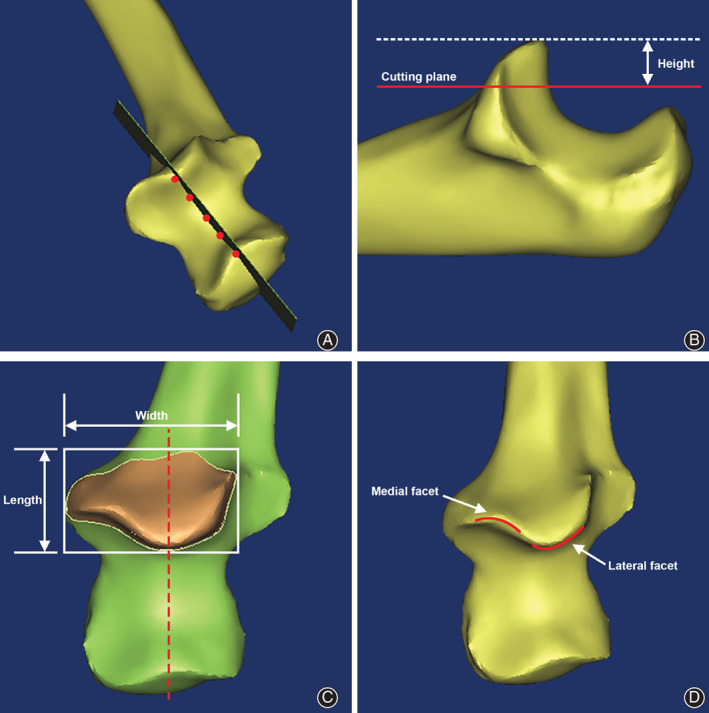
Coronoid process reconstruction and anatomical parameters definition. (A) A base plane was created passing through the points on ridge of the greater sigmoid notch (red dots). (B) A cutting plane (red line)parallel to the posterior flat spot on the olecranon was created to trim the upper 40% coronoid process and the height of the target fragment was defined. (C) The length and width of the cross section were defined. (D) Curvature of medial and lateral facet was illustrated (red curves).

To quantify the geographic characteristics of the coronoid fragment for bilateral comparison, a base plane passing through the points greater sigmoid notch was developed for the orientation of proximal ulna and subsequent measurement of parameters (Fig. 1A). A cutting plane, defined as a plane parallel to the posterior flat spot on the olecranon, was created to trim the upper 40% coronoid process for simulation of coronoid process fracture, which serves as the target fragment^6^ (Fig. 1B). The height, length, and width of target fragment as well as the radius of the medial and lateral facet were measured and compared in respect to gender, age (>40 years and < 40 years), and laterality.

### *Establishment of the Local Coordinate System*

**Fig. 2:**
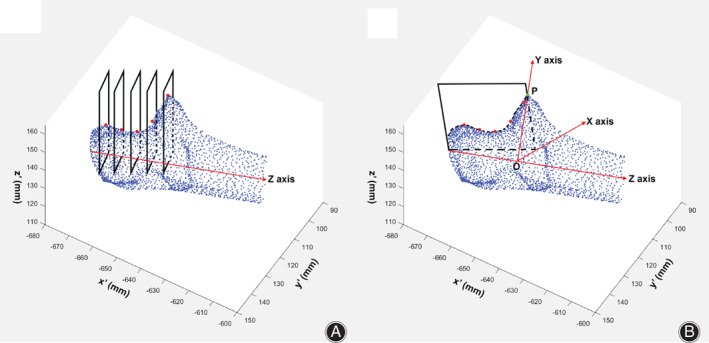
Establishment of the local coordinate system. (A) The central long axis of the proximal 20% of ulna was determined and was defined as Z axis (red line). Five points on the olecranon ridge (red dots) was determined as points with maximal Gaussian curvature by intersection of five sequential cross sections perpendicular to Z axis. (B) A plane passing the five points on the olecranon ridges (red dots) intersected the coronoid process at a tip point P (green dot). A line from the point P and perpendicular to Z axis intersected Z axis at a point O, which was defined as the origin of the local coordinate. The line from point P to point O was defined as Y axis. X axis was defined as perpendicular to both Y axis and Z axis.

The point cloud model was imported into MatLab (v7.13.0, The Math Works, Inc., Natick, MA, US) to evaluate the similarity of articular surface of bilateral coronoid and to establish a local coordinate of proximal ulna. First, the central long axis of the proximal 20% of ulna was determined by fitting coordinates of centers in three sequential cross sections of the ulna shaft, which was defined as Z axis^18^ (Fig. 2A). Second, five sequential cross sections perpendicular to the central long axis were created between olecranon tip and coronoid process with identical increments. Olecranon ridge was identified as the point with maximal Gaussian curvature on the section (Fig. 2A). A plane was generated by fitting the five points representing the greater sigmoid notch and intersecting the coronoid process at a tip point P (Fig. 2B). A line from point P and perpendicular to Z axis intersected Z axis at a point O, which was defined as the origin of the local coordinate (Fig. 2B). And the line from the point P to point O was defined as Y axis. X axis was defined as the line perpendicular to both Y axis and Z axis. A coordinate transformation algorithm was developed by reorientation of the proximal ulna with respect to the local coordinate.

### *3D* *Matching of Bilateral Coronoid Process*

**Fig. 3:**
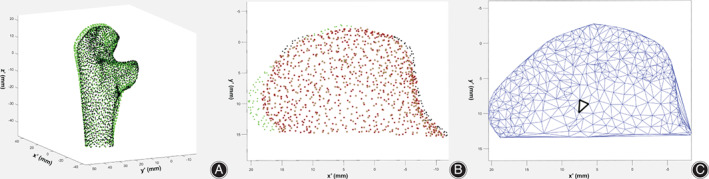
Matching of articular surface of bilateral coronoid processes and calculation of covering percentage. (A) Matching of bilateral ulna (green dots: right ulna; black dots: mirroring of left ulna). (B) Overlapping of articular surface of bilateral coronoid process (green dots: dots on the articular surface of right coronoid process more than 2 mm from the mirroring of left one; black dots: mirroring of dots on the articular surface of left coronoid more than 2 mm from the right one; black dots with red circle: dots from articular surface of bilateral coronoidprocesses within 2 mm from each other). (C) Example of Delaunay triangulation of articular surface of coronoid process for calculation of covering percentage (black triangle indicated a typical sample formed by three neighboring dots).

For 3D matching of bilateral coronoid process, the left coronoid process should be mirrored and matched to the right one in accordance with the following criteria: Z axis and the tip point on coronoid process (point A) of both ulnas should be overlapping (Fig. 3A). Based on this principle, a coordinate transformation algorithm was developed. We investigated the proximity region within a distance of 2 mm between articular surfaces of the target fragment on bilateral coronoid process (Fig. 3B). To calculate the area of the articular surface of target fragment and the proximity region in the point cloud model, Delaunay triangulation was performed on its projection on the plane formed by X axis and Y axis and total area was calculated by summation of area of each triangle^19^(Fig. 3C).

### *Radiographic Assessment*

To quantify the morphological similarity of bilateral coronoid process, the following parameters were employed for evaluation.

#### *The height of the target fragment*

The height of the target fragment (mm) is the distance from the tip of the coronoid process to the cutting plane representing 40% of the total coronoid height in the ventral to dorsal direction (Fig. 1B). This is a parameter which is important to block posterior dislocation. It was compared between different sex, age, and laterality groups.

#### *The Length of the Target Fragment*

The length of the target fragment is the distance of the fragment in the proximal to distal direction (Fig. 1C). This is to describe the proximal–distal length on the cross section of the cutting plane across the coronoid process. It was compared between different sex, age, and laterality groups.

#### *The Width of the Target Fragment*

The width of the target fragment is the distance of the fragment in medial to lateral direction (Fig. 1C). This is to describe the medial–lateral length on the cross section of the cutting plane across the coronoid process. It was compared between different sex, age, and laterality groups.

#### *The Radius of Medial Facet*

The radius of medial facet describes the curvature of the medial facet of the articular surface of the coronoid process (Fig. 1D). This is to describe the morphology of articular surface of the medial facet. It was compared between different sex, age, and laterality groups.

#### *The Radius of Lateral Facet*

The radius of lateral facet describes the curvature of the lateral facet of the articular surface of the coronoid process (Fig. 1D). This is to describe the morphology of articular surface of the lateral facet. It was compared between different sex, age, and laterality groups.

#### *Covering Percentage*

Matching of bilateral articular surface was quantified using the concept of covering percentage, which was calculated using the following equation:

$$\text{Covering percentage}=\frac{2\times S}{S_{1}+S_{2}}\times 100\%$$

In this equation, S represents the area of proximity region; S_1_ and S_2_ indicate the total area of articular surface of target fragment of left and right coronoid process, respectively. A result of 100% suggests perfect matching.

### *Statistical Analysis*

The values are expressed as the mean and standard deviation (SD). All data were evaluated with normality test. For comparison of parameters with respect to age and gender, the significant difference was quantified with independent two‐sample Student's *t*‐test, while for comparison of parameters between bilateral coronoid processes, paired two‐sample Student's *t*‐test was employed. A *P* value of <0.05 was considered significantly different. A prior measurement was conducted for calculation of the sample size for the height, width, and length of target fragment and radius of the medial and lateral facet. α and β for calculation of sample size were set to be 0.05 and 0.2. The calculated numbers for sample size for each of the above parameters were 64, 73, 82, 52, and 76 cases. In our study, a total of 128 CT scans of bilateral elbow was enough to generate a power >80%.

## Results

### *General Results*

The height, length, width as well as the radius of the medial and lateral facet of the upper 40% of the coronoid process was measured and compared. All data obeyed normal distribution (*P* = 0.67). The height of the target fragment was 12.40 ± 2.74 mm. The length of the target fragment was 23.81 ± 2.67 mm. The width of the target fragment was 23.12 ± 1.92 mm. The radius of medial and lateral facet was 6.44 ± 1.01 mm and 11.84 ± 3.71 mm, respectively.

### *Comparison of the Height of the Target Fragment*

The height of the target fragment was 12.62 ± 2.06 mm for male patients and 12.13 ± 3.76 mm for female patients. No difference was found between male and female patients (*t* = 0.94, *P* = 0.35). For comparison between different age group, the height of the target fragment was 12.79 ± 1.76 mm for patients >40 years and 13.23 ± 3.16 mm for patients <40 years. No difference was found between patients >40 years and patients <40 years (*t* = 1.11, *P* = 0.27). For bilateral comparison, the height of the target fragment of left and right coronoid process was 12.26 ± 3.40 mm and 12.74 ± 2.79 mm, respectively. No difference was found between bilateral extremities (*t* = 1.15, *P* = 0.25).

### *Comparison of the Length of the Target Fragment*

The length of the target fragment was 23.86 ± 2.11 mm for male patients and 23.76 ± 2.85 mm for female patients. No difference was found between male and female patients (*t* = 0.23, *P* = 0.82). For comparison between different age groups, the length of the target fragment was 22.92 ± 1.96 mm for patients >40 years and 23.23 ± 2.14 mm for patients <40 years. No difference was found between patients >40 years and patients <40 years (*t* = 0.76, *P* = 0.45). For bilateral comparison, the length of the target fragment of left and right coronoid process was 22.52 ± 2.89 mm and 21.66 ± 3.01 mm, respectively. No difference was found between bilateral extremities (*t* = 1.00, *P* = 0.32).

### *Comparison of the Width of the Target Fragment*

The width of the target fragment was 23.06 ± 1.54 mm for male patients and 23.19 ± 2.82 mm for female patients. No difference was found between male and female patients (*t* = 0.33, *P* = 0.74). For comparison between different age groups, the width of the target fragment was 24.82 ± 2.23 mm for patients >40 years and 23.46 ± 3.38 mm for patients <40 years. No difference was found between the two different age groups (*t* = 1.56, *P* = 0.12). For bilateral comparison, the width of target fragment of left and right coronoid process was 24.42 ± 2.22 mm and 24.47 ± 2.69 mm, respectively. No difference was found between left and right extremities (*t* = 1.31, *P* = 0.19).

### *Comparison of the Radius of Medial Facet*

The radius of medial facet was 6.41 ± 1.39 mm for male patients and 6.47 ± 0.95 mm for female patients. No difference was found between male and female patients (*t* = 0.28, *P* = 0.78). For comparison between different age groups, the radius of medial facet was 6.82 ± 1.28 mm for patients >40 years and 6.46 ± 0.94 mm for patients <40 years. No difference was found between the two different age groups (*t* = 1.31, *P* = 0.19). For bilateral comparison, the radius of medial facet of left and right coronoid process was 6.43 ± 1.24 mm and 6.64 ± 1.34 mm. No difference was found between left and right extremities (*t* = 1.60, *P* = 0.11).

### *Comparison of the Radius of Lateral Facet*

The radius of lateral facet was 11.61 ± 4.24 mm for male patients and 12.11 ± 3.09 mm for female patients (*t* = 0.74, *P* = 0.46). For comparison between different age groups, the radius of medial facet was 11.82 ± 3.28 mm for patients >40 years and 12.46 ± 3.94 mm for patients <40 years (*t* = 1.02, *P* = 0.31). For bilateral comparison, the radius of lateral facet of left and right coronoid process was 11.97 ± 5.31 mm and 10.29 ± 3.29 mm, respectively (*t* = 1.70, *P* = 0.09). No difference was found between gender, age, and bilateral extremities.

### *3D* *Matching of Bilateral Coronoid Process*

To evaluate the similarity of the bony architecture of bilateral coronoid process, especially the morphology of the articular surface, the left coronoid process was mirrored and overlapped on the corresponding right one with the covering percentage reaching 87.8% ± 12.1%. For male patients, the covering percentage was 85.3% ± 14.2%. For female patients, it was 90.0% ± 11.2%. There was no significant difference between male and female groups (*t* = 0.75, *P* = 0.41). For patients >40 years, the covering percentage was 88.2% ± 11.7%. For patient <40 years, it was 87.4% ± 13.2%. There was no significant difference between these two groups (*t* = 0.98, *P* = 0.33).

## Discussion

In this study, the anatomy of bilateral coronoid process was compared and the similarity of the articular surface was evaluated quantitatively. As hypothesized, bilateral coronoid processes share excellent similarity in the anatomy and the morphology of articular surface.

The coronoid process of ulna serves as the mainstay in the stability of elbow joint^1^. If fixation of the fracture fragment could not be achieved, reconstruction using autograft or allograft was regarded as a choice. But there was still some concern on the rarity of source and the donor site comorbidity, which resulted in the continuous investigation and development of coronoid process prosthesis to restore the function of this bony structure^7^, ^13^, ^14^, ^15^. The most important issue during the prosthesis design is to accomplish accurate restoration of the morphology of the articular surface of coronoid process in each individual case.

Three‐dimensional printing technique is suggested to better restore the complex anatomy. With the development of 3D printing technology in orthopaedics, both 3D skeletal models and implants have been put into clinical practice^20^, ^21^. The application of 3D‐printed, personalized prosthesis is a new breakthrough in 3D printing technology, which has been used to replace or restore the osseous structure or normal function. Currently, published results have suggested its application in spine, hip, knee, and elbow, which has indicated an optimistic outlook^21^, ^22^, ^23^, ^24^. It was believed that postoperative function of the joint and the longevity of the 3D printing prosthesis were closely associated with precise measurement of the anatomical parameters. As the morphological characteristics regarding the architecture of the coronoid process is missing due to comminuted fracture, spatial information from contralateral side provides a good alternative. Previous studies have suggested a high symmetry of osseous architecture of bilateral limbs, and evaluation of the pathology in one limb using the contralateral structure as a mirror‐image template has resulted in satisfactory outcome^16^, ^17^. However, results on restoration of unrepairable coronoid process defect with 3D printing technique using the information of contralateral side as template has not been reported.

Three‐dimensional printing technique depends on integration of the spatial information of the bony structure. A variety of parameters have been employed for description of the anatomical characteristic of coronoid process, which includes width, height, olecranon‐coronoid angle, articular surface area, coronoid volume^25^, ^26^, ^27^. In this study, it is confirmed that parameters such as height, length, width, radius of medial, and lateral facet could be successfully used to delineate the morphological feature of coronoid process. As the cartilage surface of the coronoid process is an intra‐articular structure, reconstruction of coronoid process requires excellent congruity to minimize the suboptimal restoration of articular surface and subsequent acceleration in development of osteoarthritis. To quantitatively evaluate the extent of matching of the articular surface of bilateral coronoid process, topographic analysis and coordinate transformation, concepts extensively used in the geographic field, were introduced. Our results suggested that bilateral coronoid process shares high similarity in morphological characteristics to articular surface.

In terms of the topographic analysis, 3D matching gained increasing popularity in the evaluation of articular surface morphology. The key step in the 3D matching is the registration between the two 3D models which depends on the identification and registration of a couple of anatomically markable points on the two 3D models. Failure in identification of the points for registration may result in systemic error. In this study, the tip of the olecranon and coronoid process, as well as the facet articulating with radial head, was extracted for registration which proved to be an easy and reliable means for the following matching.

### *Limitation*

This study had some limitations. First, the thickness of the articular cartilage was not taken into consideration as it is based on CT scan, which may compromise the evaluation of the true congruency. However, previous studies have suggested no significant difference for bilateral coronoid cartilage thickness, thus the congruency was majorly predominated by the osseous structure^28^. Second, simulation was only performed for bony defect involving the upper 40%, while the articular surface below this level was not assessed. However, considering the prevalence of coronoid process fracture as determined by the Regan–Morrey classification, most of them only involved the upper 40%, thus the result of this study was applicable in most of the clinical scenarios. Third, although the cases enrolled have already shared a similar spectrum of age with the patients diagnosed as coronoid process fracture, detailed analysis such as differences of the anatomical parameters among different ages and other factors could be further analyzed if sample size was increased.

### *Conclusions*

The present study suggested that bilateral coronoid processes share high similarity in terms of the 3D structure and articular surface morphology, which suggested that the osseous architecture of the coronoid process with comminuted fracture could be predicted by the morphological information of the contralateral side.

## Disclosure

All named authors hereby declare that they have no conflicts of interest to disclose. Ethical approval was obtained from the institutional review board (IRB) of Beijing Jishuitan Hospital and it was documented that no informed consent was required.

## Funding

This research received financial support from National Natural Science Foundation of China (NSFC 81672137).
